# Whole Exome Sequencing Identifies Novel Recurrently Mutated Genes in Patients with Splenic Marginal Zone Lymphoma

**DOI:** 10.1371/journal.pone.0083244

**Published:** 2013-12-13

**Authors:** Marina Parry, Matthew J. J. Rose-Zerilli, Jane Gibson, Sarah Ennis, Renata Walewska, Jade Forster, Helen Parker, Zadie Davis, Anne Gardiner, Andrew Collins, David G. Oscier, Jonathan C. Strefford

**Affiliations:** 1 Cancer Sciences, Faculty of Medicine, University of Southampton, Southampton, United Kingdom; 2 Human Development and Health, Faculty of Medicine, University of Southampton, Southampton, United Kingdom; 3 Department of Pathology, Royal Bournemouth Hospital, Bournemouth, United Kingdom; University of Navarra, Spain

## Abstract

The pathogenesis of splenic marginal zone lymphoma (SMZL) remains largely unknown. Recent high-throughput sequencing studies have identified recurrent mutations in key pathways, most notably *NOTCH2* mutations in >25% of patients. These studies are based on small, heterogeneous discovery cohorts, and therefore only captured a fraction of the lesions present in the SMZL genome. To identify further novel pathogenic mutations within related biochemical pathways, we applied whole exome sequencing (WES) and copy number (CN) analysis to a biologically and clinically homogeneous cohort of seven SMZL patients with 7q abnormalities and *IGHV*1-2*04 gene usage. We identified 173 somatic non-silent variants, affecting 160 distinct genes. In additional to providing independent validation of the presence of mutation in several previously reported genes (*NOTCH2, TNFAIP3, MAP3K14, MLL2* and *SPEN*), our study defined eight additional recurrently mutated genes in SMZL; these genes are *CREBBP, CBFA2T3, AMOTL1, FAT4, FBXO11, PLA2G4D, TRRAP* and *USH2A*. By integrating our WES and CN data we identified three mutated putative candidate genes targeted by 7q deletions (*CUL1, EZH2* and *FLNC*), with *FLNC* positioned within the well-characterized 7q minimally deleted region. Taken together, this work expands the reported directory of recurrently mutated cancer genes in this disease, thereby expanding our understanding of SMZL pathogenesis. Ultimately, this work will help to establish a stratified approach to care including the possibility of targeted therapy.

## Introduction

Splenic Marginal Zone Lymphoma (SMZL) is a low grade chronic B cell lymphoproliferative disorder that predominantly affects elderly patients and involves the spleen, bone marrow, and peripheral blood [1]. Although the median survival is around 10 years, approximately 70% of SMZL patients require treatment, of whom 25% experience progressive disease, leading to early death [1]. 

Our understanding of the molecular pathogenesis of SMZL remains limited. Early cytogenetic studies identified recurrent deletions of 7q31-q32 and duplications of 3q in approx. 30% and 20% of cases, respectively [2], but subsequent molecular investigations have failed to identify causative genes within these regions [3]. Candidate gene studies are limited to mutations in *TP53*, which is disrupted in 10-15% of cases [2], and to genes within the NF-ƘB pathway, which are mutated in a third of all cases [4,5]. The presence of a highly restricted immunoglobulin gene repertoire, in particular the selective usage of the immunoglobulin heavy chain variable (*IGHV*) 1-2*04 allele in 20-30% of patients, suggests that antigenic stimulation may be important in the pathogenesis of this disease [6].

The recent application of whole exome sequencing to frozen splenic tissue from 14 patients with SMZL followed by targeted resequencing of recurrent variants in larger cohorts has identified further biologically relevant genes [7,8]. Mutations in *NOTCH2*, which eliminate the C-terminal PEST domain and result in compromised protein degradation, were identified in 20 - 25% of cases although there was no consensus as to the clinical significance of these mutations between studies [7,8]. Gene mutations in modulators or other members of the Notch signalling pathway and in other pathways, such as chromatin remodelling and transcriptional regulation were also implicated [8].

In view of the relatively small number of patients investigated so far and the biological heterogeneity of SMZL, it is vital to perform additional gene discovery experiments to fully catalogue the molecular lesions that contribute to disease pathogenesis. To this aim, we performed whole exome sequencing and copy number analysis of tumour and germ-line DNA extracted from a clinically homogeneous cohort of SMZL patients. In doing so, we expand the reported directory of recurrently mutated cancer genes in this disease, thereby expanding our understanding of SMZL pathogenesis that will ultimately facilitate improvements in disease management and the promise of novel therapies.

## Materials and Methods

### Patients and biomarker analysis

Seven patients were included in this current study, all met established diagnostic criteria [1], and 5/7 underwent a splenectomy with histology typical of SMZL in each case and no evidence of transformation to a high-grade lymphoma. Each patients harboured chromosomal aberrations targeting 7q and IGHV1-2*04 usage (Table **S1**), ensuring the exclusion of other types of splenic lymphoma from our analysis and maximizing the likelihood of identifying pathogenic mutations within related biochemical pathways. Informed patient consent was obtained according to the declaration of Helsinki, and the study was ethically approved by the local REC. 

Chromosomal analysis was performed and described according to the International System for Human Cytogenetic Nomenclature [9]. Immunoglobulin variable region genes were sequenced from either cDNA or gDNA as previously described [6]. cDNA was synthesised by reverse transcription according to the manufacturers protocol (Promega). gDNA was extracted using the Qiagen Blood Mini Kit and amplified using the BIOMED 2 protocol [10]. PCR products were sequenced directly using an ABI 310 genetic analyser and sequences were aligned to the IMGT-V-Quest database. 

### High-throughput sequencing, variant calling and Sanger validation

Using targeted exome capture (SureSelect Human All Exon 51Mb V4, 50Mb V3, Agilent) we prepared sequencing libraries from high-molecular weight genomic DNA from CD19 positive-purified tumour cells (five cases extracted from the spleen and two from peripheral blood) and matched saliva cells (Oragene DNA kit, DNA Genotek) prior to high-throughput sequencing with the Illumina HiSeq system. The paired-end sequencing data were aligned against the human genome reference sequence (hg19/GRCh37) using the Novoalign software (novoalignMPI V2.08.02, Novocraft Technologies, Selangor, Malaysia). Duplicate reads, resulting from PCR clonality or optical duplicates, and reads mapping to multiple locations were excluded from downstream analysis. Depth and breadth of sequence coverage was calculated with custom scripts and the BedTools package (v2.13.2) [11] and is included in table **S2** **.**

Germ-line-Tumour paired datasets were analysed to identify single nucleotide variations (SNVs) and small insertion and deletions using Varscan 2.3.3 [12] (http://varscan.sourceforge.net). The minimum variant allele frequency threshold was set to 10% with a minimum read depth of 4. Variants were filtered using the ‘somaticFilter’ command to remove clusters of false positives and SNV calls near indels with the same frequency and depth thresholds.

Variants were annotated with respect to genes and transcripts and filtered using the Annovar software tool (v2012Jun21) [13]. Variants were cross referenced with databases of known variation were downloaded from the Annovar website (June 2012); data from the 1000 Genomes Project (2012 April release)[14], dbSNP135 (and a version with SNPs flagged as rare <1% frequency or clinically associated by NCBI) and data from 4300 European American samples from The National Heart Lung and Blood Institute Exome Sequencing Project Exome Variant Server (http://evs.gs.washington.edu/EVS/), (ESP6500 release). Using conventional Sanger sequencing, we confirmed the presence of 38/45 somatic variants (84.4%) and those non-concordant cases were due to low exome read-depth in the tumour sample.

### SNP6.0 array hybridization, data extraction and analysis

Tumour and germ-line DNA was purified, amplified, labelled and hybridized to the Affymetrix SNP6.0 platform (Affymetrix, Santa Clara, CA) as previously described [15]. For copy number analysis, two independent researchers visually inspected parallel copy number profiles (aligned to hg19/GRCh37) from tumour and germ-line samples using Partek Genomics Suite (Partek Inc, Missouri, USA), and lesions were classified as somatic if they were present and absent in the tumour and germ-line material, respectively. Copy number alterations (CNAs) were defined as a deviation of 50 consecutive array features (probes) from a normal value of 2 (±0.3), within a consecutive genomic window of 50 Kilobases. The allele ratio was calculated for each sample using the HapMap Allele Reference baseline (Affymetrix) and copy number neutral loss of heterozygosity (CNNLOH) event were defined as somatic if they were present and absent in the tumour and germ-line material, respectively. 

## Results and Discussion

Exome-capture and high-throughput sequencing allowed us to align approx. 41.9 million reads per sample at a mean depth of 69x (range, 43-109x). In total, an average of 82.2% (range, 70-95%) of target sequences captured at 20x. Our analytical pipeline identified 176 somatic non-silent variants, affecting 165 distinct genes (Table **S2**). These variants were base-pair transitions (34%), transversions (28%), insertions (6%) and deletions (31%). Copy number analysis identified 28 somatically-acquired copy number deletions (66%) and duplications (33%), (Table **S2**). Considering the mutation and copy number data together, our patients exhibited an average of 25 somatic mutations (range, 9-40) and four copy number alterations (range, 2-9) per tumour sample.

We initially investigated our exome sequencing data for the presence of somatic variants in genes known to be recurrently mutated in SMZL. In doing so, we identified mutations in *NOTCH2* [exon 34, n=2], *TNFAIP3* [n=3], *MAP3K14* [n=2], *MLL2* [n=1] and *SPEN* [n=1] (Table **1**). As the exome capture efficiency of *NOTCH2* can compromise variant identification, we also performed Sanger sequencing of exon 34 as previously reported [8]. In doing so, we found no additional mutations. Furthermore, we identified mutations in six genes that have previously been shown to harbour mutations in single SMZL cases [8] (Table **1**). This observation implicates these genes as recurrent mutational targets in SMZL. Mutations in several of these genes have been identified in other tumour types, for example *FBXO11*, which is recurrently mutated in diffuse large B-cell lymphoma (DLBCL) and promote leukaemogenesis by stabilization of BCL6 [16] (Table **1**). 

**Table 1:** Summary of recurrently mutated genes in our cases and a comparison with previously published studies.

	**Genes**	**Accession**	**Variant nomenclature**		SIFT score	Polyphen-2 score	Case no^6^.							Published
		**number**			prediction	prediction	1	2	3	4	5	6	7	Study
			Nucleotide change^4^	Amino acid change	consequences	consequences								^7^
ESTABLISHED:	***NOTCH2***	NM_024408	c.C7081T°	p.Q2361X	Damaging	Truncating					✓			[7,8]
Genes			c.6836delA§	p.H2279fs	Truncating	Truncating	✓							
recurrently	***MAP3K14***	NM_003954	c.C200G*	p. A67G	Damaging	Probably damaging					✓			[8]
mutated	***TNFAIP3***	NM_006290	c.T1343A*	p.M448K	Tolerated	Truncating						✓		[4,5,8]
in SMZL^1^			c.C1681T*	p. P561S	Tolerated	Truncating						✓		
			c.A328T*	p. T110S	Tolerated	Truncating				✓				
	***MLL2***	NM_003482	c.2507_2508insC	p.Q836fs	Truncating	Truncating						✓		[8]
	***SPEN***	NM_015001	c.C5179T°	p.Q1727X	Tolerated	Truncating						✓		[8]
			c.10286_10289del§	p.3429_3430del	Truncating	Truncating						✓		
NOVEL:	***AMOTL1***	NM_130847	c.G1270A*	p.A424T	Tolerated	Benign						✓		[8]
Recurrent	***FAT4***	NM_024582	c.G6628A*	p.A2210T	Tolerated	Benign	✓							[8]
genes across	***FBXO11***	NM_001190274	c.G1587C*	p.W529C	Damaging	Truncating				✓				[8]
studies^2^	***PLA2G4D***	NM_178034	c.23delG§	p.G8fs	Truncating	Truncating			✓					[8]
	***TRRAP***	NM_003496	c.367-10T>-^	Splicing^5^	Truncating	Truncating					✓			[8]
	***USH2A***	NM_206933	c.G7553C*	p.S2518T	Tolerated	Benign				✓				[8]
NOVEL:	***CBFA2T3***	NM_175931	c.C464T*	p.P155L	Damaging	Probably damaging					✓			Novel
recurrent			c.G1445A	p. S482N	Damaging	Probably damaging							✓	
genes in our study^3^	***CREBBP***	NM_001079846	c.A4349G*	p.Y1450C	Damaging	Truncating					✓	✓		Novel

^1^ Identifies those genes that have previously been shown to be targeted by recurrent mutations in SMZL. ^2^ Shows those genes that were mutated in single SMZL cases in both our current study and in previously published work. ^3^ Shows the novel genes targeted by recurrent mutations in our study.

^4^ Identified non-synonymous (*), splice-site (^), frameshift (§) and stopgain (°) mutations

^5^ The *TRRAP* mutation in case 5 occurred within a splice-site and is predicted to resulted in aberrant splicing

^6^ Showed the presence (✓) and absence (white box) of each mutation in the patients in our series

^7^ Highlights the published studies that identified the mutations in each of the genes listed

Next we investigated our SMZL cases for recurrent mutations in genes that have not been previously identified in SMZL (Table **1**). This analysis identified two genes, *CREBBP* and *CBFA2T3*, both mutated in two patients, which in the context of the published literature provides a potential prevalence of approx. 10% in SMZL. Both of the *CREBBP* mutations were the Y1412C variant previously identified in DLBCL [17]. *CREBBP* is involved in chromatin remodelling and transcription factor recognition, and this mutation has been shown to compromise the protein’s ability to acetylate BCL6 and p53 [17]. The *CBFA2T3* gene, a core binding factor from the myeloid translocation gene family, is targeted by recurrent chromosomal rearrangements in both lymphoid and myeloid malignancies. Whilst non-synonymous in nature, our mutations were not located within the key ETO, MTG16 or TAFH functional domains of the protein. In pediatric B-cell lymphoma, *CBFA2T3* has been implicated as a cellular proto-oncogene as in rare cases the gene is juxtaposed to the immunoglobulin locus [18]. In AML chromosomal inversions involving *CBFA2T3* can directly increase the self-renewal capacity of hematopoietic progenitors [19]. Mutations in both these genes were present in approx. 50% of reads, suggesting they are heterozygous mutations present in the dominant tumour clone.

To further assess the potential biological impact of the mutations observed in our cases, pathway analysis was performed using the Database for Annotation, Visualisation and Integrated Discovery (DAVID) (Table **2**). In addition to identifying pathways already implicated in SMZL pathogenesis, such as notch signalling (*NOTCH2*, *NOTCH4*), we also show that genes within MAPK signalling pathway are targeted by somatic non-synonymous mutations in the majority of our cases (5/7, 71%). Whilst a biological role of these genes in SMZL required functional confirmation, our data does suggest that the MAPK signalling pathway is a major target for somatic mutations in this sub-group of SMZL. 

**Table 2:** Summary of the pathways in which mutated genes in our SMZL cohort can be found and their predicted functional consequences.

**DAVID Pathway**	**Genes**	**Accession numbers**	**Variant nomenclature**		**SIFT score prediction**	**Polyphen-2 score prediction**	**Case no^2^.**						
			**Nucleotide change**	**Amino acid change**	**consequences**	**consequences**	**1**	**2**	**3**	**4**	**5**	**6**	**7**
**MAP kinase**	*CACNA1E*	NM_001205293	c.G1069C	p.E357Q	Damaging	Damaging				✓			
	*CACNA1H*	NM_021098	c.391delG	p.E131fs	Truncating	Truncating			✓				
	*CACNA2D2*	NM_001174051	c.2837delC	p.P946fs	Truncating	Truncating		✓					
	*FLNC*	NM_001458	c.C3179T	p.P1060L	Damaging	Probably damaging					✓		
	*MAP3K14*	NM_003954	c.C200G	p. A67G	Truncating	Truncating				✓	✓		
	*MAPK8IP3*	NM_001040439	c.743delA	p.Q248fs	Truncating	Truncating		✓					
	*RASA1*	NM_002890	c.C142A	p. P48T	Damaging	Truncating						✓	
	*TAOK3*	NM_016281	c.438-7-T)	Splicing^1^	Truncating	Truncating		✓					
**Notch**	*NOTCH2*	NM_024408	c.C7081T	p.Q2361X	Truncating	Truncating					✓		
			c.6836delA	p.H2279fs	Truncating	Truncating	✓						
	*PIWIL3*	NM_001008496	c.2242delA	p.T748fs	Truncating	Truncating		✓					
	*NOTCH4*	NM_004557	c.C5877G	p.C1959W	Truncating	Damaging						✓	
	*MAML3*	NM_018717	c.1513_1514del	p. 505_505del	Truncating	Truncating						✓	
**Cell cycle**	*CUL1*	NM_003592	c.T469G	p.Y157D	Damaging	Probably damaging				✓			
	*CREBBP*	NM_001079846	c.A4349G	p.Y1450C	Damaging	Truncating				✓		✓	
	*CDC27*	NM_001114091	c.A701C	p. Y234S	Tolerated	Benign						✓	
**Cytokine-cytokine**	*FLT1*	NM_002019	c.2594_splice	splicing	Truncating	Truncating	✓						
**receptor interaction**	*CRLF2*	NM_022148	c.G340C	p.V114L	Tolerated	Probably damaging				✓			

^1^ The *TRRAP* mutation in case 5 occurred within a splice-site and is predicted to resulted in aberrant splicing

^2^ Showed the presence (✓) and absence (white box) of each mutation in the patients in our series

Finally, we identified somatically acquired mutations in genes also targeted by 7q deletions in our patients. In doing so, we found *CUL1*, *FLNC* and *EZH2* mutations in individual cases (Table **2**). Of these gene mutations, only *FLNC* was located within the published 7q MDR [3,20]. *FLNC* mutations have not been previously identified in a series eight del(7q) cases [20], suggesting that the prevalence of *FLNC* mutation is low in this sub-type of SMZL. However, further research will be required to establish if rare mutations represent only one mechanism of gene deregulation, as repression of *FLNC* transcription by promoter methylation in prevalent in several other human cancer types [21-24]. The somatic variant we identified in *EZH2* (p.K199N), which is located outside the SET protein domain, is not the activating mutation prevalent in follicular lymphoma and DLBCL [25] nor has it been previously reported in AML or MDS [26].

Herein, for the first time, we report the analysis of a homogeneous cohort of SMZL cases using whole exome sequencing and copy number analysis. In doing so, we validate the presence of recurrent mutations in several genes with established importance in SMZL. Furthermore, we expand the reported directory of recurrently mutation cancer genes in this disease, with the most significant observation being the identification of recurrent mutations in *CREBBP* and *CBFA2T3*. The importance of *CREBBP* is further strengthen by the presence of a single SMZL case in the literature with a small deletion that juxtaposes 16 exons of *CREBBP* with the *ZNF434* gene, resulting in loss of the acetyltransferase domain of the CREBBP protein [8]. Furthermore, we show the majority of cases in our series carried mutations within MAPK signalling genes, suggesting that mutations in these genes are strongly associated with 7q-rearranged SMZL with IGHV1-2*04 usage. Whilst our analysis identifies a series of novel genes mutated in SMZL, a larger study is required to determine the frequency of these events and any utility in the risk-adapted stratification of SZML patients. To this aim, we are currently coordinating a pan-European study into the presence of somatic mutations in approx. 750 genes with a known or postulated role in SMZL pathophysiology in a cohort of more than 300 SMZL cases. This will ultimately establish the frequency and clinical importance of gene mutations in SMZL and help to establish a stratified approach to care including the possibility of targeted therapy. 

## Supporting Information

Table S1 **Clinical characteristics of each patient included in the study.**
(DOCX)

Table S2 **Shows the 176 non-silent somatic mutations identified in our cases.**
(XLSX)
