# Supplementary material for: Whole Exome Sequencing Identifies Novel Recurrently Mutated Genes in Patients with Splenic Marginal Zone Lymphoma
Source: PLoS One. 2013 Dec 13;8(12):e83244. doi: 10.1371/journal.pone.0083244 (PMC3862727; doi:10.1371/journal.pone.0083244)
Supplement: Table S1 — Clinical characteristics of each patient included in the study. (DOCX) [file pone.0083244.s001.docx]

**Supplementary Table 1. Patient characteristics**

| Case no. | **Local RegID** | **Gender** | **Age at Diagnosis (yrs)** | **% Tumor Cells** | **ISCN Karyotype** | ***IGHV* gene usage** |
| --- | --- | --- | --- | --- | --- | --- |
| 1 | 363 | M | 75 | 80 | 47,XY,del(11)(q23q25),+12/46,XY,del(7)(q32q34) | 1-02*04 |
| 2 | 365 | F | 76 | 68 | 46,XX,del(7)(q32q34) | 1-02*04 |
| 3 | 366 | F | 47 | 51 | 46,XX,der(7)t(3;7)(?;q32) | 1-02*04 |
| 4 | 367 | F | 54 | 70 | 46,XX,del(7)(q22q36) | 1-02*04 |
| 5 | 452 | F | 49 | 74 | 46,XX,del(7)(q31.2) | 1-02*04 |
| 6 | 437 | F | 64 | - | 46,XX,del(7)(q34q36) | 1-02*04 |
| 7 | 453 | M | 84 | 81 | 46,XY,inv(7)(q22q23),del(6)(q21q24) | 1-02*04 |

Tumour cell percentage was not available for patient 6
